# A time-course study of long term over-expression of ARR19 in mice

**DOI:** 10.1038/srep13014

**Published:** 2015-08-11

**Authors:** Imteyaz Qamar, Mohammad Faiz Ahmad, Arul Narayanasamy

**Affiliations:** 1 School of Biotechnology, Gautam Buddha University, Greater Noida-201308, India; 2 School of Biotechnology, Jawaharlal Nehru University, New Delhi-110067, India; 3 Department of Life Science, Research Center for Cell Homeostasis, Ewha Womens University, Seoul 120-750, Republic of Korea

## Abstract

A leucine-rich protein, ARR19 (androgen receptor corepressor-19 kDa), is highly expressed in male reproductive organs and moderately in others. Previously, we have reported that ARR19 is differentially expressed in adult Leydig cells during the testis development and inhibits steroidogenesis by reducing the expression of steroidogenic enzymes. Whereas in prostate, ARR19 represses the transcriptional activity of AR (androgen receptor), it is important for male sexual differentiation and maturation in prostate and epididymis, through the recruitment of HDAC4. In this study we show that long term adenovirus mediated overexpression of ARR19 in mice testis has the potential of inhibiting the differentiation of testicular and prostatic cells by reducing the size of testis and prostate but has no effect on the growth of seminal vesicles. Further, it reduces the level of progesterone and testosterone by reducing the steroidogenic enzymes such as 3HSD, P450c17 and StAR. This is the first study reporting a time-course analysis of the implications of long term overexpression of ARR19 in mice testis and its effect on other organs such as prostate and seminal vesicles. Taken together, these results suggest that ARR19 may play an important role in the differentiation of male reproductive organs such as testis and prostate.

The steroid hormone testosterone plays an important role in the development and function of male reproductive organs, controlled by the hypothalamic-pituitary-gonadal axis. The gonadotropin-releasing hormone (GnRH), produced by the hypothalamus, stimulates production and secretion of luteinizing hormone (LH) and follicle stimulating hormone (FSH) by pituitary gonadotrope cells. LH then stimulates Leydig cell testosterone synthesis by binding to its G- protein-coupled receptor (LHR) leading to increased cAMP production and ultimately activation of steroidogenesis^1^. Testicular steroidogenesis in adult leydig cells is primarily regulated by the gonadotropin luteinizing hormone (LH) through the production of the intracellular second messenger cAMP. LH/cAMP stimulates steroidogenesis^2^ by increasing the expression of several steroidogenic enzymes such cytochrome P450 protein 17, the steroid 21-hydroxylase, and 20-α-hydroxysteroid dehydrogenase^3^,^4^,^5^,^6^,^7^,^8^.

ARR19 (androgen receptor corepressor-19 kDa), which is also known as chemokine-like factor superfamily 2a (*Cklfsf2a)*, belongs to a group of novel proteins that operate as a functional bridge between chemokines and members of the transmembrane 4 superfamily. Members of the CKLFSF family have been suggested to perform an important role across a broad range of physiological and pathological processes. In human, CKLFSF2 is expressed abundantly in the testis and has two counterparts in the mouse, Cklfsf2a and Cklfsf2b. Cklfsf2a and Cklfsf2b have similar expression patterns and amino acid identities of 47.6 and 45.5%, respectively, with human CKLFSF2^9^. Mouse Cklfsf2a/ARR19 was originally cloned as a potential androgen response gene in the testis and was further characterized as a novel androgen receptor (AR) corepressor^10^. The ARR19 gene encoding a hydrophobic leucine-rich 19-kDa protein is abundantly expressed in the testis and moderately in other male reproductive organs such as the prostate^9^,^10^.

Previously we reported that ARR19 is highly expressed in the early stages of Leydig cell development, with progressively less expression at later stages. The expression of the ARR19 gene is regulated by LH/cAMP signaling via the GATA-1 transcription factor together with cAMP response element-binding protein-binding protein^11^,^12^. ARR19 acts as an anti-steroidogenic factor, physically interacts with Nur77 and inhibits testicular steroidogenesis by suppressing the Nur77-induced expression of steroidogenic enzyme genes^11^,^12^. However, these studies monitored only short term effects of ARR19.

In the present study, we carry this work forward and present the implications of long term over-expression of ARR19 in mice testis in a time-dependent manner and its impact on the development of testis and other organs such as prostate and epidydimis. For this, whole organismal study was conducted in mice. Our results present a time-course analysis of ARR19 overexpression, for upto 14-days in mice, and its implications on the levels of testosterone, progesterone and the steroidogenic enzymes in the isolated Leydig cells of mice. We find that as the levels of ARR19 increase, the levels of the steroidal enzymes and the steroidogenic enzymes decrease, and this inhibition was observed to be maintained upto a period of 14 days of ARR19 overexpression. Upon analyzing the organ weights from ARR19 overexpressing mice, we further found that ARR19 significantly reduced the weight of testis and prostate compared to the control mice, which indicates the inhibition of differentiation of testicular and prostatic cells. However the molecular mechanism behind the inhibition of prostatic cells by overexpression of ARR19 in testis is currently under consideration. These finding suggest that ARR19 plays a vital role in the differentiation of reproductive organs such as testis and prostate.

## Materials and Methods

### Animals and Treatment

6 week old FVB mice were purchased from commercial supplier (Daehan Laboratories, Daejeon, Korea). The selection of mouse ages was based on previous report of the development of adult Leydig cells^13^. Animals were sacrificed by CO_2_ asphyxiation, and testes were extracted for several analyses. Evaluation of testis weight, Western blot analysis and Radioimmunoassay, 6 week old mouse testes were infected with 5 × 10^7^ particles of recombinant adenovirus harboring green fluorescent protein (Ad-GFP) or ARR19 (Ad-ARR19) in a phosphate-buffered saline (0.01 M, pH 7.2) for defined time periods. Adenovirus was delivered to testes under a dissecting microscope using glass micropipettes. All animal procedures and experimental protocol were approved by Institutional Animal Ethics Committee and followed the standards set forth in the Guide for the Care and Use of Laboratory Animals (National Academy of Science, Washington, D.C.). All efforts were made to minimize the animal suffering and to reduce the number of the animals used. The methods were carried out in accordance with the approved guidelines.

### Measurement of testes, prostate and seminiferous tubules weight

Mice were weighed and sacrificed, after 0, 3, 7, 10 and 14 days of ARR19 overexpression. The various organs, (testes, prostate and seminiferous tubules), were isolated and weighed.

### Purification of Primary Leydig Cells

Purification of mouse Leydig cells was carried out as described previously^14^ with some modifications. The animals were anesthetized prior to decapitation. Six testis/set (0, 3, 7, 10, 14 hrs of infection) were removed, and the testicular cells were dispersed by treating the decapsulated testes with collagenase (0.25 mg/ml; Sigma-Aldrich) in M199 medium, and the solution was filtered. Interstitial cells were precipitated by centrifugation of the filtrate and washed once with M199 and twice with phosphate-buffered saline^15^.

### Preparation of Recombinant Adenovirus

For the ectopic expression of mouse ARR19, an adenoviral delivery system was used^16^. Briefly, HA-tagged ARR19 cDNA was cloned into pAdTrack-CMV shuttle vector. Homologous recombination was performed by transforming adEasy-BJ5138-competent cells with pAdTrack-CMV-ARR19 together with adenoviral gene carrier vector. The recombinant viruses were selected, amplified in HEK-293 cells, and purified cesium chloride density centrifugation. The number of virus particle was calculated by measuring optical density at 260 nm (A_260_) as previously reported^11^.

### Adenovirus injection

The mice were anesthetized by i.p. injection of the ketaset cocktail (0.1 ml per 100 g body weight) and the lower abdominal portion was cleaned with ethanol and shaved with a razor. The shaved area was further cleaned by gently swabbing with ethanol followed by Triadine. The intact testes were slightly exposed by excision and the lower abdominal skin of mice near the testis was opened by using scalpel. The injection needle containing 5 × 10^7^ virus particles/testis in PBS were injected manually in a location between the seminiferous tubules using a stationary 10-ml syringe connected to glass capillary injection needle. Then injected testis were carefully placed back into the abdominal cavity and the abdominal wall (sutured) and skin (wound clips) of live animal were surgically closed.

### Western Blot Analysis

The cell lysates were prepared in radioimmuno precipitation assay cell lysis buffer (50 mM Tris-HCl, pH 8.0, 100 mM NaCl, 1% Nonidet P-40, 5 mM EDTA, pH 8.0, 1 mM Na_3_VO_4,_ 1 mM Na_2_P_2_O_7_, 1 g/ml aprotinin, 0.1 g/ml leupeptin, 1 g/ml pepstanin, and 0.1 mM phenylmethysulfonyl fluoride) and were separated via SDS-PAGE. The protein were transferred onto nitrocellulose transfer membranes and were subsequently subjected to Western blot analysis using anti-ARR19, anti-P450c17, anti-3*β* HSD, anti-StAR, or anti-actin (Santa Cruz Biotechnology, Inc.) antibody. The signals were the visualized using ECL kit (Amersham Biosciences). Actin signals were used as a loading control.

### Radioimmunoassay

The testosterone and progesterone concentrations were measured by RIA. The testes were isolated from 6-week-old mice after the infection with Ad-ARR19 or Ad-GFP at various time points (0, 3, 7, 10, 14 days). The dissected testes were homogenised in phosphate-buffered saline, and steroids were extracted three times with volumes of diethyl ether. The experiment was repeated three times, and the assay procedures were followed as described previously^17^.

### Immunohistochemistry

The testis were fixed in 4% paraformaldehyde and embedded in paraffin. Tissue sections (4–6 μm) were subjected to immunohistochemistry in accordance with standard procedure (11) using rabbit anti-ARR19. A horseradish peroxidase-streptavidin histostain-Plus (Zymed, S. San Francisco, CA) system was employed to visualize the signals. The samples were analyzed via light and phase-contrast microscopy (Leica DMRXA microscope; Leica AG, Heerbrugg, Switzerland).

### Statistical Significance

Statistical significance was calculated using Student’s *t* test. The *p* values are indicated by ‘*’ in the figures with the following levels of significance: *p ≤ 0.05; **p ≤ 0.01; ***p ≤ 0.001; ****p ≤ 0.0001. All *p* values less than 0.0001 are indicated by ****.

## Results and Discussion

### ARR19 inhibits the level of testosterone of mouse testis

**Figure 1:**
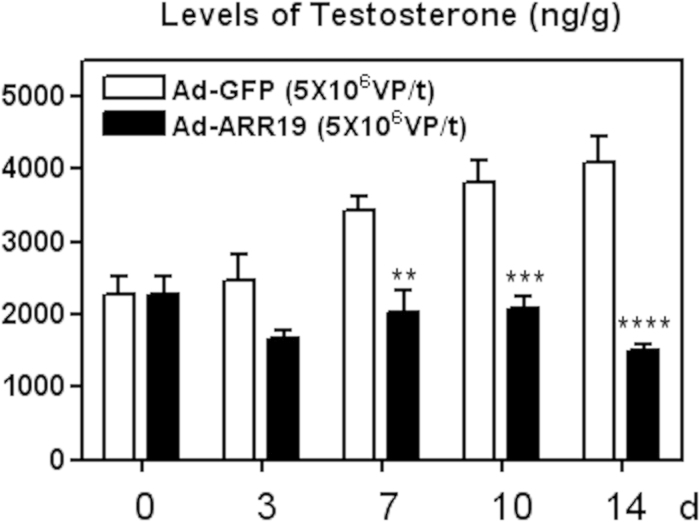
ARR19 inhibits the level of testosterone of mouse testis. Testis of three 6-week old adult mice were infected with Ad-ARR19 or control Ad-GFP at a concentration of 5 × 10^7^ virus particle/testis and were dissected out after 0, 3, 7, 10 and 14 days of experiment. Testis extract were subjected to RIA to measure testicular testosterone levels. The testosterone levels of both the groups, (Ad-ARR19 & Ad-GFP), were compared after 0, 3, 7, 10 and 14 days of infection. The levels of testosterone showed significant reduction after 3 days of ARR19 overexpression as compared to the control group (p value for: 0 days = 1; 3days = 0.06; 7 days = 0.002; 10 days = 0.0005; 14 days = 0.00003). This result thus suggested an inhibitory role of ARR19 on the testosterone levels in mice. The experiment was repeated three times, each time with three mice.

Previous studies have shown that short term adenovirus-mediated overexpression of ARR19 in the dissected testis inhibits the biosynthesis of steroidal hormones (11, 12) in 6-week old mice. However, could the overexpression of ARR19 continue the inhibition of steroids for longer time or does the effect diminish after a few days. To address this question, we initially attempted to test Adenovirus mediated overexpression system in mouse testis and the testicular regions/locations that overexpress ARR19 after infection with Ad-ARR19. Immunohistochemical analyses were conducted using testis sections prepared after 0 and 14 days of Ad-ARR19 infection. As expected, the expression of ARR19 was detected in interstitial regions between seminiferous tubules, which are mainly populated by leydig cells in mouse testis (Supplemental Fig. 1). Next, we set an experiment in which we infected the mice testis with Ad-ARR19, and, with Ad-GFP as a control. We then analyzed the levels of testosterone using testicular extract till 2 weeks (Fig. 1). Interestingly, we found that the levels of testosterone gradually increased in the control mice, increasing from 0–14 days after infection with the control Ad-GFP. However, in mice overexpressing ARR19, the levels of testosterone displayed comparable and quite significant decrease as compared to the control. The levels of testosterone were observed to be decreased after 3-days of ARR19 overexpression and it continued to decrease further after 7, 10 and 14 days. The inhibition of ARR19 on days 7, 10 and 14 was more significant (p = 0.002; 0.0005; and 0.00003 respectively), as compared to inhibition at 3 days (p = 0.06), when compared with their controls. Thus, a long term overexpression of ARR19 could maintain a suppressed level of testosterone in the testicular Leydig cells, having profound implications on the differentiation and functioning of the testicular Leydig cells.

### ARR19 inhibits the level of progesterone of mouse testis

**Figure 2:**
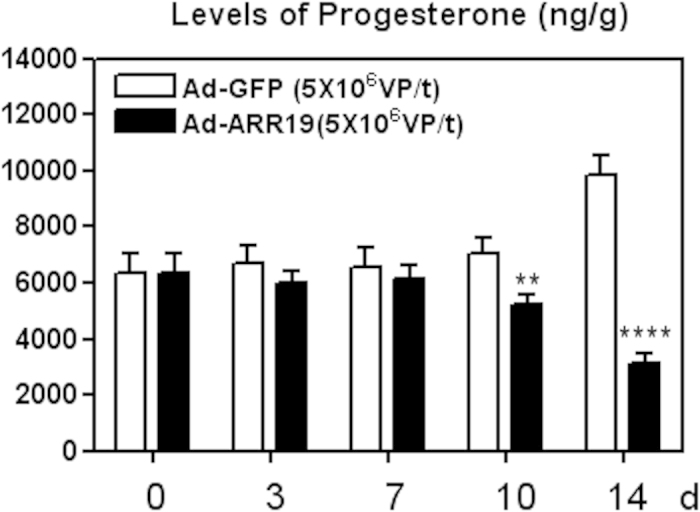
ARR19 inhibits the level of progesterone of mouse testis. Testis of three 6-week old adult mice were infected with Ad-ARR19 or control Ad-GFP at a concentration of 5 × 10^7^ virus particle/testis and were dissected out after 0, 3, 7, 10 and 14 days of experiment. Testis extract were subjected to RIA to measure testicular progesterone levels. The progesterone levels were compared for both the groups, (Ad-ARR19 & Ad-GFP), after 0, 3, 7, 10 and 14 days of infection. The levels of progesterone showed reduction upon ARR19 overexpression, as compared to the control group being significant at 10 and 14 days of ARR19 overexpression (p value for: 0 days = 1; 3 days = 0.3; 7 days = 0.6; 10 days = 0.01; 14 days = 0.000007), thus suggesting an inhibitory role of ARR19 on the progesterone levels in mice. The experiment was repeated three times, each time with three mice.

We next analyzed the effect of long term overexpression of ARR19 upon the levels of the progesterone levels in the mice testis (Fig. 2). We observed that the levels of progesterone upon ARR19 overexpression were quite similar to the control Ad-GFP upto 7 days of infection, in contrast with the inhibition of testosterone starting from day 3. However, on the 10^th^ day we observed a significant decrease in the progesterone levels in the mice overexpressing ARR19 (p = 0.01). The levels were found to be further decreased after 14 days (p = 0.000007). Interestingly, the levels of progesterone in control were also similar after 0 to 10 days of Ad-GFP infection, which was different from level of testosterone observed. Thus, long term overexpression of ARR19 in the testis of mice has the potential of decreasing the progesterone levels also, similarly to the reduction in testosterone levels. However, the effects of ARR19 overexpression on the testosterone level appear to be more immediate as compared to the implications on the progesterone levels.

### ARR19 inhibits the steroidogenic enzyme genes in mouse testis

**Figure 3:**
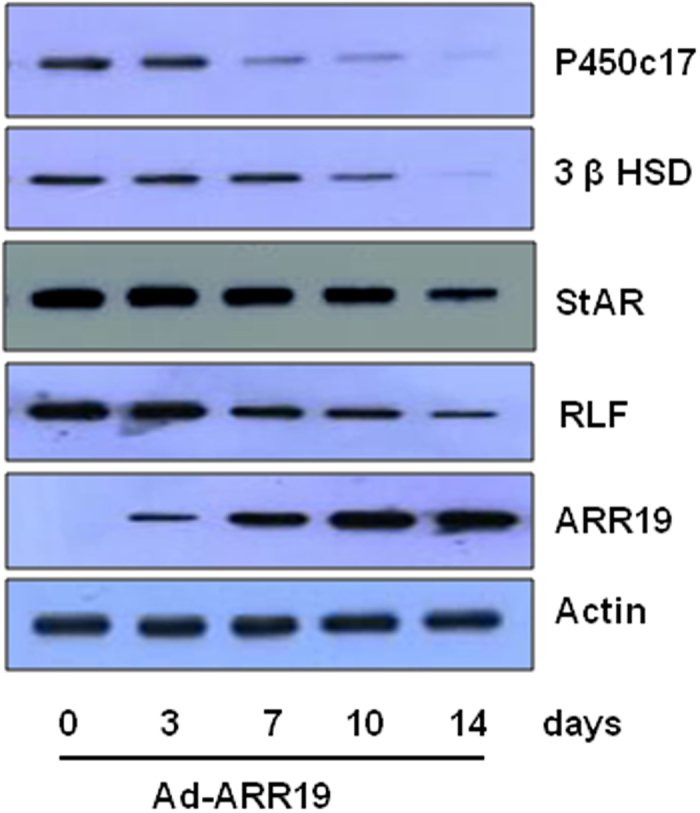
ARR19 inhibits the levels of steroidogenic enzymes in mouse testis. Three 6-week old mice testis were infected with Ad-ARR19 at a concentration of 5 × 10^7^ virus particle/testis and were subjected to isolate testicular Leydig cells after different time intervals (0, 3, 7, 10 & 14 days) post-infection. Proteins from the isolated testicular Leydig cells were isolated and analyzed using Western blot analysis. The levels of ARR19 increased gradually after 3 days of infection. However, as the levels of ARR19 increased the levels of the steroidogenic enzymes P450c17, 3 β HSD, StAR and RLF decreased suggesting an inhibitory role of ARR19 on the levels of steroidogenic enzymes in mice. The experiment was repeated three times, each time with three mice. The gels have been run under the same experimental conditions.

Steroidogenesis in testicular Leydig cells is regulated by LH/cAMP mediated expression of steroidogenic enzymes genes such 3 β HSD, p450 c17, StAR etc. To confirm the inhibitory effect of overexpression of ARR19 in steroids production, we next analyzed the expression of steroidogenic enzymes using Western blot analysis (Fig. 3). The proteins from the purified testicular Leydig cells of mice were analyzed after 0, 3, 7, 10 and 14 days of Ad-ARR19 infection. Actin levels were taken as control and were found to be similar in all cases. The ARR19 expression was observed from 3^rd^ day post-infection of mice, which confirms the presence of ARR19 protein. ARR19 showed a further increase on 7^th^ day and showed a further increase on the 10^th^ day post-infection. The levels were found to be similar on the 10^th^ and 14^th^ day, suggesting a stabilized and sustained expression of ARR19. However the levels of the steroidogenic enzymes genes 3 β HSD, p450 c17, StAR continuously decreased as the levels of ARR19 increased. The enzyme P450c17 levels were significantly reduced after 7 days of overexpression of ARR19, and almost diminished by the 14^th^ day. The levels of 3 β HSD were significantly reduced after 10 days of overexpression of ARR19, and almost completely abrogated by the 14^th^ day. The levels of StAR also showed observable reduction after 14 days of overexpression of ARR19. Importantly, the enzyme Relaxin-like factor, also known as Leydig cell insulin like factor which is important for Leydig cell differentiation^18^,^19^,^20^, showed reduced levels after 7 days of ARR19 overexpression, and more reduced levels after 14 days of ARR19 overexpression. Thus, these results suggest that ARR19 plays a crucial role is the control of steroid production in Leydig cells through the regulation of steroidogenic enzyme gene expression

### ARR19 inhibits the steroidogenic enzyme genes in mouse testis

**Figure 4:**
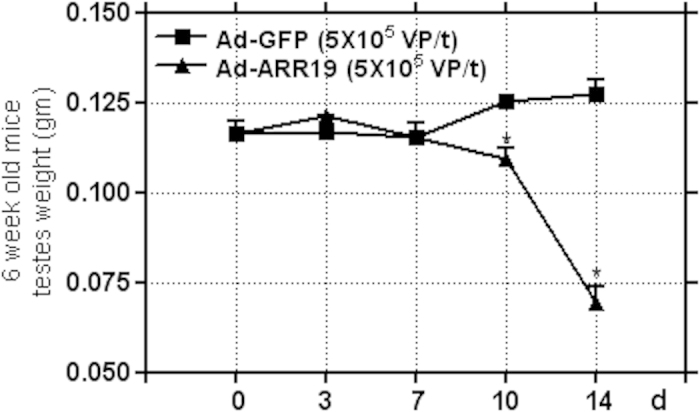
ARR19 inhibits the steroidogenic enzyme genes in mouse testis. Three 6-week old mice testis were infected with Ad-ARR19 at a concentration of 5 × 10^7^ virus particle/testis and Ad-GFP as control. The testes from both the groups of mice were isolated after 0, 3, 7, 10 and 14 days of infection, and analyzed for size and weight. No difference in testes weight was observed in both the groups, upto 7 days of Ad-ARR19 infection. However, the testes weight showed reduction after 10 days of ARR19 overexpression (p = 0.03), and was significantly reduced after 14 days of infection (p = 0.02), as compared to the control group, suggesting an important role of ARR19 on the differentiation of testicular cells in mice. The experiment was repeated three times, each time with three mice.

To study the role of ARR19 in the differentiation of mice testicular cells, we isolated and analyzed the weight of mice testes after different time intervals upon overexpression of ARR19, and compared it with the control group of mice (Fig. 4). We found that the testes weight between the control and the experimental group was similar till 7 days after overexpression of ARR19. However, the testis weight reduced significantly after 10 days of overexpression of ARR19 (p = 0.03), and we observed a further sharp decrease after 14 days of ARR19 overexpression (p = 0.02). The reduced testicular weight upon ARR19 overexpression suggests that ARR19 overexpression inhibits the differentiation of testicular cells and thus lead to testicular atrophy.

### Effect on growth of mouse prostate by ARR19 infection

**Figure 5:**
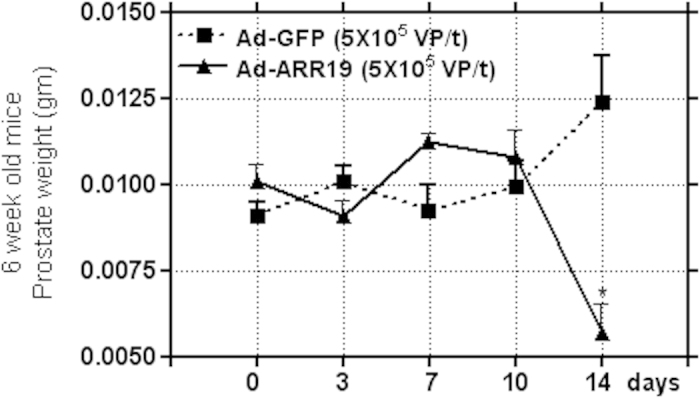
Effect on growth of mouse prostate by ARR19 infection. Three 6-week old mice testis were infected with Ad-ARR19 at a concentration of 5 × 10^7^ virus particle/testis and Ad-GFP as control. The prostate from both the groups of mice were isolated after 0, 3, 7, 10 and 14 days of infection, and analyzed for their weight. No difference in weight was observed in both the groups upto 10 days of infection (Ad-ARR19 or Ad-GFP). However, the prostate growth was largely reduced after 14 days (p = 0.02), suggesting the inhibition of ARR19 infection on the differentiation of prostatic cells in mice compare to control Ad-GFP. The experiment was repeated three times, each time with three mice.

We next analyzed how the overexpression of ARR19 affected the differentiation of prostatic cells. We again compared the weight of prostate from mice infected with Ad-ARR19, after different time intervals and compared it with mice form the control group (Fig. 5). We did not see any significant difference in the weight of prostate from the two groups, upto 10 days after ARR19 overexpression. However, after 14 days of ARR19 overexpression, we found a marked decrease in prostate weight, as compared to the control group, thus suggesting an influence of ARR19 in the differentiation of prostatic cells (p = 0.02).

### Effect on growth of mouse seminal vesicles by ARR19 infection

**Figure 6:**
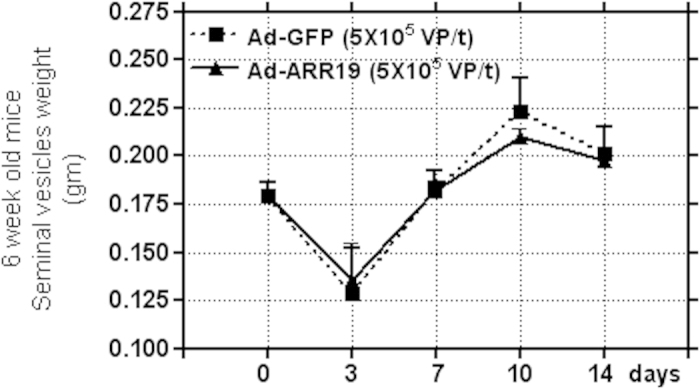
Effect on growth of mouse seminal vesicles by ARR19 infection. Three 6-week old mice testis were infected with Ad-ARR19 at a concentration of 5 × 10^7^ virus particle/testis for overexpression of ARR19 and Ad-GFP vector as a control. The seminal vesicles from both the groups of mice were isolated after 0, 3, 7, 10 and 14 days of ARR19 overexpression, and analyzed for their weight. No difference in weight was observed, suggesting no influence of ARR19 on the differentiation of seminal vesicles in mice. The experiment was repeated three times, each time with three mice.

We also analyzed how ARR19 overexpression could affect the growth of seminal vesicles. For this, we compared the weight of the seminal vesicles isolated from the mice overexpressing ARR19, and compared it to the control group (Fig. 6). We did not observe any difference in the weight of seminal vesicles isolated from both the groups, even after 14 days of overexpression of ARR19, thus suggesting that ARR19 does not influence the growth of seminal vesicles in mice.

## Conclusions

In this study we have analyzed the long term role of overexpression of an anti-steroidogenic factor, ARR19 in various organs such as testis, prostate and seminal vesicles of mice. Also we have studied the effect of overexpression of ARR19 in the process of steroidogenesis through the production of testosterone and progesterone. Our studies clearly show the suppression of the levels of testosterone and progesterone by ARR19 through the inhibition of steroidogenic enzymes such as StAR, 3 β HSD, and p450c17. Importantly, a key marker of leydig cell differentiation, which is known as Relaxin like factor, is also reduced by ARR19 overexpression. These results suggest that ARR19 may play an important role in the differentiation of testicular Leydig cells. Apart from testis, we found that the size of prostate is also significantly reduced by ARR19 overexpression. This could be because ARR19 represses the transcriptional activity of Androgen Receptor (AR) through recruitment of histone deacetylase in prostate as reported previously (10). However it would be interesting to know how the testicular infection by ARR19 affects the differentiation of prostatic cell in mice. Taken together, the study suggests that ARR19 may act as an important factor for cellular differentiation, not only in testis but also for other organs such as prostate.

## Additional Information

**How to cite this article**: Qamar, I. *et al.* A time-course study of long term over-expression of ARR19 in mice. *Sci. Rep.* **5**, 13014; doi: 10.1038/srep13014 (2015).

## Supplementary Material

Supplementary Figure 1
