# Supplementary material for: A time-course study of long term over-expression of ARR19 in mice
Source: Sci Rep. 2015 Aug 11;5:13014. doi: 10.1038/srep13014 (PMC4531322; doi:10.1038/srep13014)

A time-course study of long term over-expression of ARR19 in mice

Imteyaz Qamar[1#]*, Mohammad Faiz Ahmad[2#] and Arul Narayanasamy[3]

[1]School of Biotechnology, Gautam Buddha University, Greater Noida-201308, India
[2]School of Biotechnology, Jawaharlal Nehru University, New Delhi-110067, India
[3]Department of Life Science, Research Center for Cell Homeostasis, Ewha Womens University, Seoul 120-750, Republic of Korea

[#] Equal Contributing Authors

[*]To whom correspondence should be addressed: Dr. Imteyaz Qamar, School of Biotechnology, Gautam Buddha University, Greater Noida, Gautam Budh Nagar, U.P.-201308, India, Tel: +91-120-2344280; E-mail: imteyazqamar@gmail.com

Supplementary Figure

Supplemental Figure 1: Adenovirus mediated overexpression of ARR19 expression mouse testis. ARR19 expression was detected in the interstitial compartment of 6-week old mice testis infected with Ad-ARR19 at a concentration of 5×10^7 virus particle/testis, after 14 days. The arrowheads point to the cells that express ARR19 within interstitial region of seminiferous tubules. 2°Ab+H: immuno-cytochemistry with the secondary antibody; (2°Ab): a negative control along with nuclear staining with hematoxylene (H).

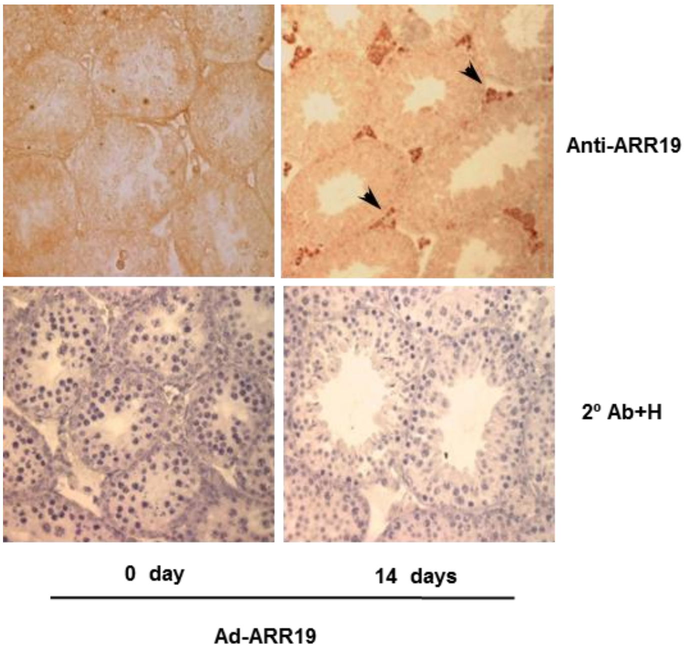

Supplement: Supplementary Figure 1 [file srep13014-s1.pdf]
